# Case Report: Tadpole pupil and concurrent migraine in an adolescent patient is a novel correlation

**DOI:** 10.3389/fped.2024.1446691

**Published:** 2024-10-08

**Authors:** Alexander Scheid, Rebecca Lise Gammelgaard Henneberg, Jonas Kjeldbjerg Hansen

**Affiliations:** ^1^ Department of Pediatrics and Adolescent Medicine, Aalborg University Hospital, Aalborg, Denmark; ^2^ Department of Clinical Medicine, Aalborg University, Aalborg, Denmark

**Keywords:** case report, tadpole pupil, vestibular migraine, pediatric ophthalmology, pediatric neurology

## Abstract

**Background:**

Tadpole pupil is a rare phenomenon characterized by a brief and irregular deformation of the pupil caused by segmental contraction of the iris dilator muscle. It is most prevalent in adult women and is, in these cases, often associated with migraine.

**Case presentation:**

We present a unique case of a 16-year-old girl who presented with recurrent episodes of tadpole pupil and vestibular migraine. This association has not been previously demonstrated in pediatric patients. During a thorough clinical examination, a thyroid carcinoma was found which due to its localization was not causative of the tadpole pupil and was considered an incidental finding.

**Conclusions:**

The association between migraine and tadpole pupil in this patient, which has not previously been described in pediatric patients, adds to the demographics of tadpole pupil. A possible pathophysiological link between the two conditions is discussed but further research is needed to understand the pathophysiology underpinning it.

## Background

Tadpole pupil (TP) is a rare phenomenon with only a few cases described. It is defined by episodic, irregular, and usually unilateral distortion of the pupil caused by segmental contraction of the dilator iris muscle, lasting from seconds to minutes and occurring in clusters. Additional symptoms may include a “strange sensation” in the affected eye and blurred vision (1).

Two case series have described TP in greater detail and, currently, 43 cases have been reported in the literature. TP is most often observed in women aged between 22 and 48 years old. Two patients had associated Horner syndrome ipsilateral to their TP (2).

In one study, a correlation between a tadpole pupil and migraine was found in 8/26 patients and a possible migraine in 3/26 (1). A switch of sides between episodes was only observed in 23% of the cases. The shape of the tadpole pupil can either be constant by involving the same segment or different with various segments of the iris being involved (2). Only three pediatric cases have been reported and all three appeared to be caused by particular events (2).

We present a case of TP in a 16-year-old girl with concurrent migraine. This is, to our knowledge, the first published pediatric case in which this association is described. Thus, the presented case provides new insight into pediatric TP and specific considerations regarding pediatric TP are discussed.

## Case presentation

**Figure 1:**
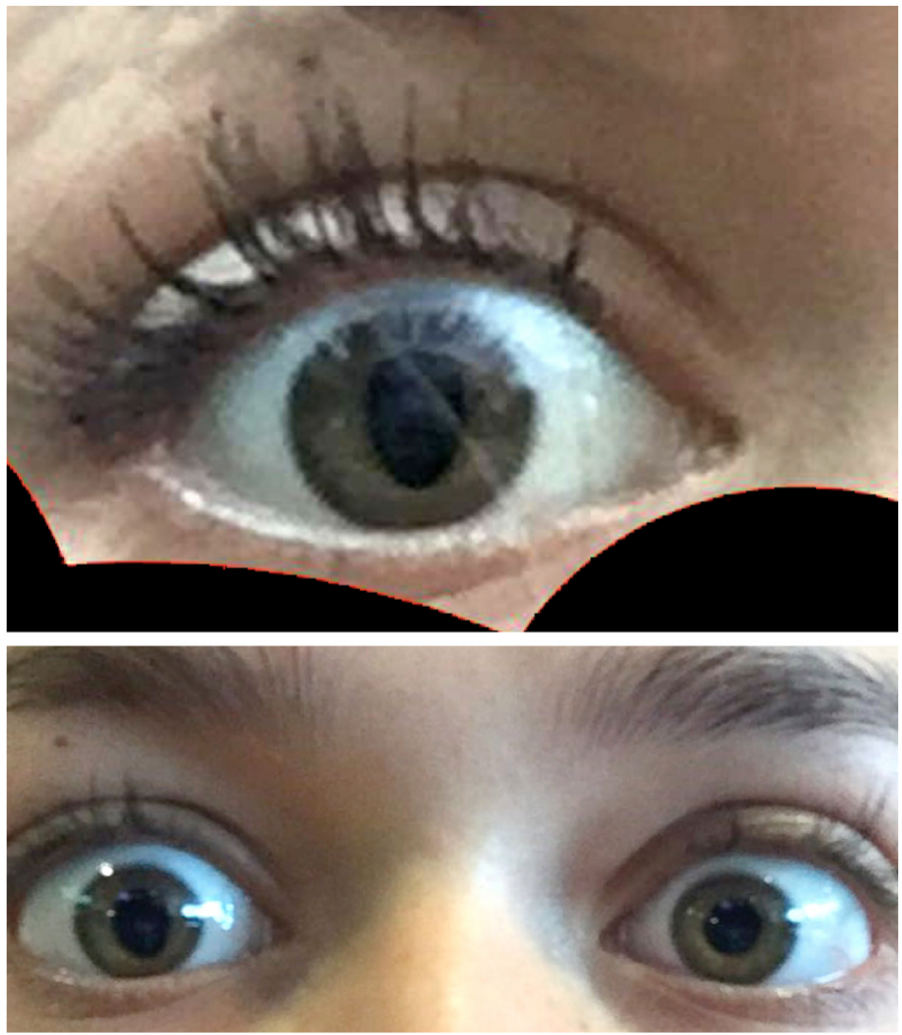
Photographs (taken by the patient) showing a right-sided tadpole pupil.

A 16-year-old girl presented with episodic deformation of the right pupil at intervals of 14 days to 3 months over 2 years. The individual episodes lasted a few seconds but often occurred in clusters with numerous episodes on the same day. The pupil usually assumed a teardrop shape (Figure 1) but could also become line-shaped.

Physical examination, including palpation of the neck and abdomen and a complete neurological examination, was normal.

An ophthalmological examination was conducted while the pupil was normal. It showed normal vision and ophthalmoscopy, a normal anterior chamber of the eye, and the topical apraclonidine test was normal. The diagnosis of a TP was made.

All blood tests were normal (thyroid levels, calcium, electrolytes, white blood cell count, platelets, hemoglobin, kidney and liver function, vitamin D and B12, and blood glucose and hemoglobin A1C). Urine catecholamine metabolites (homovanillic acid and vanillylmandelic acid) were in the normal range.

**Figure 2:**
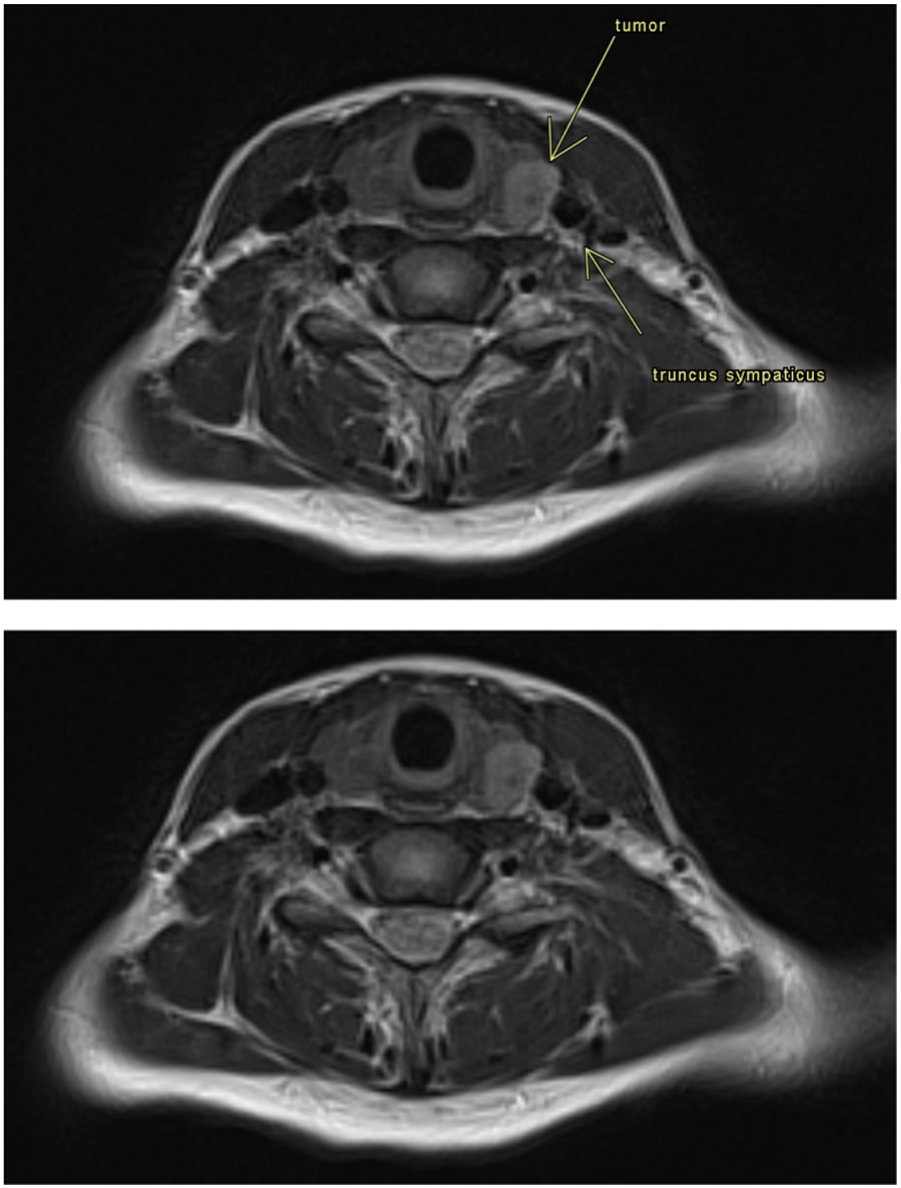
MRI was done on an Avanto Fit 1.5T (Siemens, Erlangen). The image is a transverse T2-weighted sequence with a thickness of 6 mm. The following were described by a neuroradiologist consultant: The image shows a tumor in the thyroid gland on the left side. There is no influence of the truncus sympathicus.

MRI scans of the chest, neck, and cerebrum, the latter including angiography, showed a 1.2 cm × 1.4 cm large process in the left thyroid lobe traveling distally between the trachea and the common carotid artery to the aortic arch, continuing into the anterior mediastinum (Figure 2). Two lymph nodes were seen on the left side of the neck. Apart from this, the MRI, including the size of the thyroid gland, was normal, and no compression of the sympathetic nerve was found.

A total thyroidectomy and extirpation of the cervical lymph nodes was performed. On histological examination, two papillary adenocarcinomas were found in the thyroid gland (one in the left lobe, one in the isthmus) and metastasis in 11 of 31 lymph nodes, all from the left side of the neck.

Thyroxine replacement therapy was instituted, and the patient was submitted to radioactive iodine therapy.

Both of the patient's parents had migraine. She, too, suffered from migraine attacks with aura (bilateral scotoma) with varying frequency. Furthermore, she experienced attacks of rotatory vertigo and nausea sometimes followed by migraine headaches. A diagnostic workup included orthostatic blood pressure and heart rate and an electrocardiogram (ECG). The patient underwent 6 days of ambulatory ECG monitoring, during which numerous episodes of vertigo occurred, and a complete ear, nose, and throat examination. All tests were normal. It was concluded that she suffered from vestibular migraine, as all other causes of vertigo were excluded.

The TP and the migraine and vertigo attacks would often occur together.

A trial treatment with lamotrigine resulted in almost complete remission of both the vertigo and headaches but did not affect the tadpole pupil.

The patient has currently been without relapse of the thyroid cancer for 4.5 years but continues to experience TP albeit at a lower frequency than at the initial referral time.

## Discussion and conclusion

Due to the correlation between Horner syndrome and TP, we chose, in this case, to follow a suggested procedure for the evaluation of pediatric TP to rule out compression of the sympathetic chain. A thyroid carcinoma contralateral to the TP was found. If there was a link between this and the TP, ipsilateral carcinoma and TP would have been expected. Furthermore, the MRI scans showed no compression of the sympathetic chain. Finally, the TP continued to occur during 4.5 years of follow-up after the patient was declared free of cancer. Thus, we concluded that there was no relation between the carcinoma and the TP.

The presented case correlates well with already described cases of TP with recurring episodes of TP lasting for a few seconds, sometimes occurring in clusters. The TP often occurred together with migraine headaches and was not provoked by any other factors. As aforementioned, TP and migraine often occur concurrently in adult patients, mainly women, but this association has not previously been described in pediatric patients with TP.

The presented patient was diagnosed with vestibular migraine. Specific diagnostic tests such as videonystagmography, video head impulse, caloric tests, or subjective virtual verticality to further evaluate her vertigo were not performed. This is a limitation of this study and we recommend that these tests be performed in similar cases. Nevertheless, the diagnostic workup was sufficient to conclude that the diagnostic criteria for vestibular migraine were fulfilled according to the definition formulated by the Committee for the Classification of Vestibular Disorders of the Bárány Society and the Migraine Classification Subcommittee of the International Headache Society (IHS) (3). Data on the use of prophylactic medication against migraine in pediatric and adolescent patients is sparse. According to open-label studies showing the effect of lamotrigine on vestibular migraine, we chose to treat the patient with lamotrigine which had a good effect on both migraine frequency and vestibular symptoms (4).

Different theories on the pathophysiology of TP have been proposed. Thompson et al. considered segmental denervation hypersensitivity to be the cause of TP (1). Another consideration was whether circulating hormones have the capacity to activate the iris dilator muscle, thus attempting to explain the higher occurrence of TP in menstruating women and the variation in shape and peak in different episodes. The same authors discussed the possibility of spontaneous and segmental oculosympathetic neuronal discharge (2).

With regard to pediatric cases, they appear to be caused by particular events. A non-complicated strabismus surgery on a 2-year-old boy elicited a single episode of tadpole pupil lasting for only 45 min (5). Furthermore, a 2-year-old girl was observed to have recurrent episodes of TP in her right eye for 40 min after waking up. The authors hypothesized that peak morning cortisol was a possible explanation (6).

Bilateral TP lasting up to 20 min in association with physical exercise was described in a 12-year-old girl. This might support Thompson et al.’s hypothesis since the authors suggested a focal hypersensitivity to circulating catecholamines to be the pathophysiological mechanism (7). In the two latter cases, the reproducibility of TP provided a reason to hypothesize that there are different pathophysiological mechanisms among children (3/4).

A case report described another episodic pupillary disorder, benign episodic unilateral mydriasis (BEUM), which has both demographic and clinical similarities with TP. Autonomic dysfunction is well described in patients with migraine and it is striking that both BEUM and TP respectively occur concurrently in a large proportion of the cases (8).

Could the high percentage of TP patients with concurrent migraine be explained by this autonomic dysfunction? The exact pathophysiology remains unknown, and the presented case does not provide definite evidence for the pathophysiology behind TP. We suggest there may be more than one possible pathophysiological explanation for the occurrence of TP.

In conclusion, a tadpole pupil is a rare phenomenon, especially in pediatric patients. The presented case is the first published pediatric case with TP and concurrent migraine and thus it provides new insight into the phenotype of pediatric TP.

At present, there is no consensus on the necessary diagnostic evaluation of pediatric TP due to the lack of publications and evidence. More knowledge about pediatric TP, including the pathophysiology, is needed and we urge researchers to publish similar cases.

## Data Availability

The original contributions presented in the study are included in the article/Supplementary Material, further inquiries can be directed to the corresponding author.
